# Efficacy and Safety of *Panax ginseng* Sprout Extract in Subjective Memory Impairment: A Randomized, Double-Blind, Placebo-Controlled Clinical Trial

**DOI:** 10.3390/nu16121952

**Published:** 2024-06-19

**Authors:** Hyang-Im Baek, Ki-Chan Ha, Yu-Kyung Park, Tae-Young Kim, Soo-Jung Park

**Affiliations:** 1 Department of Food Science & Nutrition, Woosuk University, Wanju 55338, Republic of Korea; hyangim100@gmail.com; 2 Healthcare Claims & Management Inc., Jeonju 54858, Republic of Korea; omphalos9121@hanmail.net (K.-C.H.); yukyungpark07@gmail.com (Y.-K.P.); 3 BTC Corporation, Ansan 15588, Republic of Korea; tykim@btcbio.com; 4 Department of Sasang Constitutional Medicine, College of Korean Medicine, Woosuk University, Jeonju 55338, Republic of Korea

**Keywords:** sprout ginseng, smart farming, subjective memory impairment, memory improvement, functional food, clinical trial

## Abstract

Sprout ginseng extract (ThinkGIN™) manufactured through a smart farm system has been shown to improve memory in preclinical studies. This study conducted a 12-week randomized, double-blind, placebo-controlled clinical trial to evaluate the efficacy and safety of ThinkGIN™ for improving memory in subjective memory impairment (SMI). Subjects aged 55 to 75 years with SMI participated in this study. A total of 80 subjects who met the inclusion/exclusion criteria were assigned to the ThinkGIN™ group (*n* = 40, 450 mg ThinkGIN™/day) or a placebo group (*n* = 40). Efficacy and safety evaluations were conducted before intervention and at 12 weeks after intervention. As a result of 12 weeks of ThinkGIN™ intake, significant differences in SVLT, RCFT, MoCA-K, PSQI-K, and AChE were observed between the two groups. Safety evaluation (AEs, laboratory tests, vital signs, and electrocardiogram) revealed that ThinkGIN™ was safe with no clinically significant changes. Therefore, ThinkGIN™ has the potential to be used as a functional food to improve memory.

## 1. Introduction

Population aging means that the proportion of elderly population increases while that of the young population decreases [1]. Population aging is a global phenomenon. According to projections of the World Population Prospects by the United Nations (UN), the elderly are expected to make up 16% of the world’s population by 2050 [2]. As the population ages, rates of age-related health problems, including dementia due to memory deficits and cognitive decline, are expected to increase [3]. Alzheimer’s disease (AD) is an age-related progressive neurodegenerative disease. It is the most common cause of dementia. AD has a significant impact on individuals and society. Its prevalence is increasing as the elderly population increases [4]. Therefore, AD is recognized as a global public health priority by the World Health Organization (WHO) [4,5].

When a normal person progresses to AD due to aging or other causes, they go through three stages [6,7]. The first stage is subjective memory impairment (SMI), in which objective memory tests are within normal ranges. However, the individual subjectively complains of impaired memory. The second stage is mild cognitive impairment (MCI), in which cognitive functions, especially memory, are reduced compared to people of the same age. This is an intermediate stage between normal aging and dementia. The third stage is the dementia stage, where SMI and MCI become AD over time without proper management or treatment. AD includes a variety of behavioral changes, including memory impairment, difficulty remembering names and phone numbers, language difficulties, decreased visual and spatial abilities, and emotional changes [8,9]. Currently, there are no effective pharmaceutical treatments for AD (i.e., treatments that have been proven to alter the underlying disease pathology or disease process) [4]. Additionally, drug treatment can be accompanied by various side effects, including nausea and dizziness [10]. This emphasizes the importance of preventing memory deficits and cognitive decline in SMI and MCI stages [3]. Therefore, it is necessary to develop safe, effective functional foods that can improve SMI and MCI stages.

*Panax ginseng*, also called Korean ginseng, is a representative traditional herbal medicine that has been widely used in Korea and China for a long time to treat various diseases [11]. *P. ginseng* can improve blood circulation, cognition, and memory with mental strengthening, immune enhancing, anti-diabetic, anti-aging, and anti-cancer effects [12,13,14]. Therefore, it is currently consumed as a popular health functional food and used worldwide as a natural medicine [15].

*P. ginseng* is known to contain a variety of active compounds, including ginsenosides, polysaccharides, amino acids, volatile oils, and polyacetylenes. In particular, ginsenosides as representative ingredients of *P. ginseng* are known to have neuroprotective effects by regulating synaptic plasticity, cholinergic system, amyloid β (Aβ) aggravation, tau hyperphosphorylation, anti-neuroinflammation, antioxidants, and anti-apoptosis [15,16]. However, the use of ginseng has problems such as its high price, long cultivation period, and quality changes depending on the natural environment. For this reason, sprout ginseng that can be grown quickly while increasing the content of ginsenosides has recently attracted interest [17,18,19,20]. Ginseng sprouts have a shorter cultivation period than ginseng. They are recognized as a crop with high economic efficiency and potential value [21,22].

Smart farming of sustainable production facilities can utilize information communication technologies to improve plant quality and productivity by providing accuracy, efficient use of resources, and optimal conditions [23,24]. Sprout ginseng grown by smart farming has high economic and potential value because of its short cultivation period and year-round cultivation [25,26,27]. In addition, cultivation conditions can be precisely controlled without being affected by the external environment, providing uniform, clean, and pesticide-free plants [27,28]. Additionally, the contents of ginsenosides (Rb2, Rb1, Rc, Rd, and Re) in sprout ginseng are increased, as shown in previous studies [17,18,21].

According to a previous study [17], sprout ginseng extract (ThinkGIN™) produced through smart farming improved cognitive and memory deficits by regulating AKT/ERK/CREB signaling in a scopolamine-induced memory deficit mouse model. In addition, it showed an effect of improving neuroinflammation through regulation of NO, iNOS, and COX-2 production in LPS-induced BV-2 microglia. These results might be due to anti-inflammatory effects of ginsenosides increased through smart farming [17].

Although the memory improvement effect of ThinkGIN™ has been confirmed through in vitro and in vivo studies, clinical trials are needed to confirm its efficacy in humans. Therefore, we conducted a 12-week, randomized, double-blind, placebo-controlled clinical trial to evaluate the memory improvement effectiveness and safety of ThinkGIN™ in SMI.

## 2. Materials and Methods

### 2.1. Ethics Statement

All subjects participated in the study after signing an informed consent form. This study was reviewed and approved by the Institutional Review Board (IRB) of Woosuk University Korean Medicine Hospital (IRB approval No.: WSOH IRB H2203-02; approved 21 March 2022) and registered with the Clinical Research Information Service (CRIS), the Republic of Korea (http://cris.nih.go.kr, registration number: KCT0007225; Registered 25 April 2022). Our clinical trial was conducted in accordance with the Declaration of Helsinki and Korean Good Clinical Practice (KGCP) guidelines. This trial was conducted according to Consolidated Standards of Reporting Trials (CONSORT) guidelines for randomized clinical trials.

### 2.2. Study Participants

A total of 80 subjects with SMI participated in this clinical trial. Inclusion criteria were as follows: (1) males and females aged 55 to 75 years; (2) subjects complaining of subjective memory impairment (Subjective Memory Complaints Questionnaire (SMCQ) score of 1 or more); (3) subjects showed a decrease of more than 1 standard deviation (SD) of age and educational level standards in one or more of the three items (free recall, delayed recall, recognition test) of the Seoul Verbal Learning Test (SVLT) or Rey Complex Figure Test (RCFT), which are memory tests [29,30]; and (4) those who provided written consent after being thoroughly educated about the study’s aims and goals. Those who had cognitive impairment due to brain disease or mental illness and those who scored 29 to 63 (severe) on the Beck Depression Inventory (BDI) were excluded. Those who were judged by the principal investigator to be inappropriate for participation in this study because of laboratory test results, and so on, were also excluded.

### 2.3. Study Design and Randomization

This was a 12-week, single-center, randomized, double-blind, parallel group, placebo-controlled clinical trial to evaluate the effectiveness and safety of ThinkGIN™ in improving memory. This study was conducted at Woosuk University Korean Medicine Hospital from March 2022 to December 2022.

Subjects had a first visit within 4 weeks of the screening visit to review their suitability based on inclusion and exclusion criteria. Subjects (*n* = 80) who met the inclusion and exclusion criteria were randomly assigned in a 1:1 random manner to the ThinkGIN™ group (*n* = 40) or the placebo group (*n* = 40). Randomization was based on random A and B number sequences generated in the randomization module of the SAS^®^ system version 9.4 (SAS Institute, Cary, NC, USA). Codes were generated before the start of this study. During the study period, all research investigators and subjects remained double-blinded from the randomization code.

Subjects were required to make a total of 4 visits, including 3 visits (visit 1: week 0; visit 2: week 6; and visit 3: week 12) after the screening visit. Baseline data were measured at the first visit (week 0). Subsequent visits were conducted at intervals of every 6 weeks.

Dietary intake and physical activity were recorded at baseline and the end of study. Subjects maintained dietary habits, life styles, and physical activities as usual during the study period. Changes in dietary intake were assessed through dietary records. Dietary intake was analyzed by a dietitian using a computer-aided nutritional analysis program (CAN-pro, Korean Nutrition Society, Seoul, Republic of Korea). Physical activity was assessed with metabolic equivalent (MET) value using the global physical activity questionnaire (GPAQ).

### 2.4. Study Products and Interventions

Study products used in this study were provided by BTC (Ansan, Republic of Korea). The preparation method was described in a previous study [17]. Briefly, sprout ginseng produced under a smart farming system was dried to be used as raw material. Dried ginseng sprouts were extracted with 20 times the volume (*v*/*v*) of 50% ethanol for 4 h at 80 °C, filtered, and concentrated to prepare ThinkGIN™.

The daily intake dose was calculated based on preclinical trials [17,31]. The ThinkGIN™ group was administered 2 capsules after breakfast for a total of 700 mg (containing 450 mg ThinkGIN™) per day. Similarly, the placebo group also took 2 capsules after breakfast for a total of 700 mg (without ThinkGIN™) orally per day. Placebo products were manufactured with ingredients that did not affect effectiveness. They were mainly composed of crystalline cellulose. ThinkGIN™ and placebo products had the same appearance, weight, and properties.

### 2.5. High-Performance Liquid Chromatography (HPLC) Analysis

**Figure 1:**
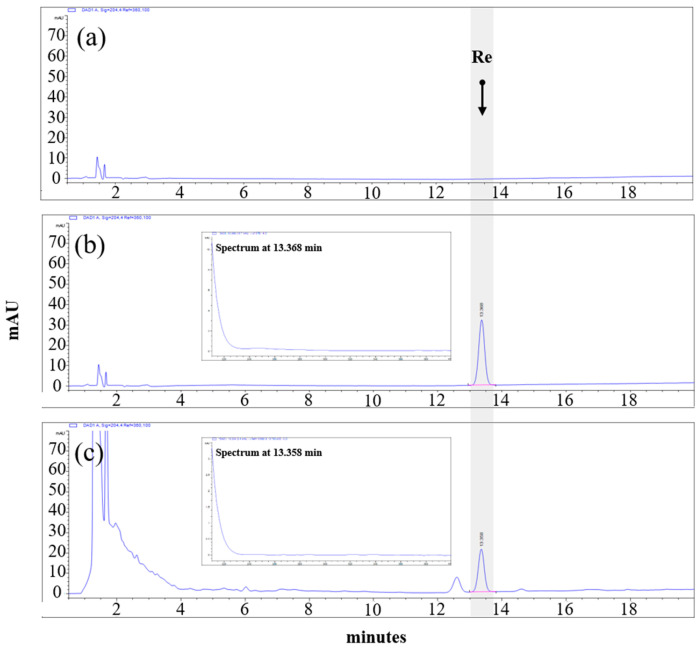
HPLC chromatographic profiles of (**a**) blank, (**b**) ginsenoside Re standard (**c**) ThinkGIN™ with excitation at 204 nm.

HPLC analysis was applied to determine the ginsenoside Re content of the ThinkGIN™ using an Agilent Infinity 1200 series system with a diode array detector (Agilent Technologies, Palo Alto, CA, USA). The separation was performed at 25 °C using a SUPELCO Discovery^®^ C18 column (4.6 mm × 250 mm, 5 μm, Merck KGaA, Darmstadt, Germany). The mobile phase was composed of distilled water and acetonitrile. The gradient dilution conditions were as follows: 0–10 min, 20% acetonitrile; 10–20 min, 20–30% acetonitrile; 20–22 min, 30–70% acetonitrile; 22–29 min, 70–100% acetonitrile. The flow rate was 1.6 mL/min and the injection volume was 5 μL. Chromatograms were obtained at a wavelength of 204 nm. The ginsenoside Re concentration of the ThinkGIN™ was standardized to 26.5~39.7 mg/g, and the HPLC chromatogram of the ThinkGIN™ is shown in Figure 1.

### 2.6. Efficacy Outcome Measures

Primary outcomes were SVLT, RCFT, and Korean version of the Montreal Cognitive Assessment (MoCA-K; total score, sub-scores). Secondary outcomes were SMCQ, Korean version of the Pittsburgh Sleep Quality Index (PSQI-K), and blood markers (acetylcholinesterase (AChE), brain-derived neurotrophic factor (BDNF), Aβ, ApoE4, total antioxidant status (TAS), and high-sensitivity C-reactive protein (hs-CRP)).

These outcomes were measured before and 12 weeks after intervention. Verbal memory was assessed through SVLT. Immediate recall was performed three times. In addition, 20-min delayed recall and recognition tests were conducted [32]. Visuospatial construction ability and visual memory ability were assessed with RCFT. Copy, immediate recall, delayed recall, and recognition tests were conducted [33]. Cognitive function was assessed using MoCA-K, which consisted of a total score and sub-scores (visuospatial-executive, naming, attention, language, abstraction, delayed recall, and orientation). MoCA-K scores range from 0 to 30, with higher scores indicating better cognition, and scores above 23 indicating normal [34].

SMCQ is a self-report questionnaire that measures the severity of memory impairment subjectively experienced by the subject. It consists of 14 questions measuring memory impairment in general and daily living. The higher the score, the more severe the decline in cognition and memory. SMCQ was verified and applied to Koreans [35]. PSQI-K is a self-report questionnaire that evaluates sleep quality. It is composed of 7 components (subjective sleep quality, sleep latency, sleep duration, habitual sleep efficiency, sleep disturbances, use of sleeping medication, and daytime dysfunction) with 19 items. The total score of PSQI-K ranges from 0 to 21, with a higher score indicating a poorer sleep quality. A total score exceeding 5 is defined as a poor sleeper. The PSQI-K is a reliable and valid questionnaire for evaluating sleep quality [36].

To measure blood biomarkers related to memory, blood samples were collected after a 12 h overnight fast. To measure plasma Aβ in blood, blood (3 mL) was collected into an EDTA tube and mixed by shaking more than 10 times immediately after sampling. Blood samples were centrifuged at 3000 rpm for 10 min. The supernatant was stored in the freezer (−70 °C) and analyzed. Aβ 40 and Aβ 42 protein levels in plasma samples were measured using the Human Amyloid Beta Assay kit (IBL, Gunma, Japan), a commercial enzyme-linked immunosorbent assay (ELISA) kit. For analysis of serum biomarkers (AChE, BDNF, ApoE4, TAS, hs-CRP), blood (5 mL) was collected into an SST tube, left at room temperature for 30 min for clotting, and then centrifuged at 3000 rpm for 10 min. The supernatant was stored in the freezer (−70 °C) and then analyzed. For serum AChE analysis, a Human Acetylcholinesterase/ACHE ELISA Kit (Novus Biological, Littleton, CO, USA) was used. BDNF was analyzed using a Human ELISA Kit Quantikine (R&D Systems, Minneapolis, MN, USA). ApoE4 was analyzed using a MILLIPLEX^®^ Human Neuroscience Magnetic Bead Panel 2—Neuroscience Multiplex Assay (Millipore, Billerica, MA, USA). TAS was analyzed using a Total Antioxidant Status Assay Kit (Mega Tıp, Gaziantep, Turkey). Serum hs-CRP concentration was measured by particle-enhanced turbidimetry (Roche Diagnostics, Mannheim, Germany).

### 2.7. Safety Outcome Measures

To evaluate safety in this study, all adverse events (AEs) that occurred during the study period were monitored. Laboratory tests, vital signs, physical examination, and electrocardiogram were performed before and at 12 weeks after study participation. Blood was collected after fasting for 12 h. Laboratory tests included complete blood count (CBC) (hemoglobin, hematocrit, white blood cell (WBC), red blood cell (RBC), and platelets], biochemistry (AST, ALT, gamma-glutamyl transferase (gamma-GT), total cholesterol, low-density lipoprotein-cholesterol (LDL-C), high-density lipoprotein-cholesterol (HDL-C), albumin, total protein, total bilirubin, alkaline phosphatase (ALP), lactate dehydrogenase (LD), glucose, blood urea nitrogen (BUN), triglyceride, creatinine, and creatine kinase (CK)], and urinalysis (pH and specific gravity). Vital signs including systolic blood pressure (SBP), diastolic blood pressure (DBP), and pulse were measured at each visit.

### 2.8. Statistical Analysis

The sample size was calculated based on the change in MoCA-K before and after intervention in ThinkGIN™ and placebo groups. A power calculation was applied by referring to a similar previous study [37]. The sample size was calculated to be a total of 80 subjects (40 subjects per group), considering a dropout rate of 30%. Therefore, 80 subjects were enrolled for 1:1 randomization to ThinkGIN™ and placebo groups.

All statistical analyses were performed using SAS^®^ version 9.4 (SAS Institute, Cary, NC, USA). Continuous variables are presented as means ± SD and categorical variables are presented as numbers (percentage). Statistical analysis of effectiveness was mainly performed on the per-protocol (PP) set, with the intent-to-treat (ITT) set additionally analyzed. The safety evaluation was performed with the safety set, which included those who participated in the study and consumed the study product at least once.

Comparison between groups was performed using an independent *t*-test of change values (12 weeks after intake—before intake). Comparison within each group was performed using a paired *t*-test of changes between before and 12 weeks after intake. Analysis of covariance (ANCOVA) was performed by adjusting for covariates when baseline characteristics were statistically significantly different between groups. Differences were considered statistically significant when the *p*-value was less than 0.05.

## 3. Results

### 3.1. Subject Characteristics

**Figure 2:**
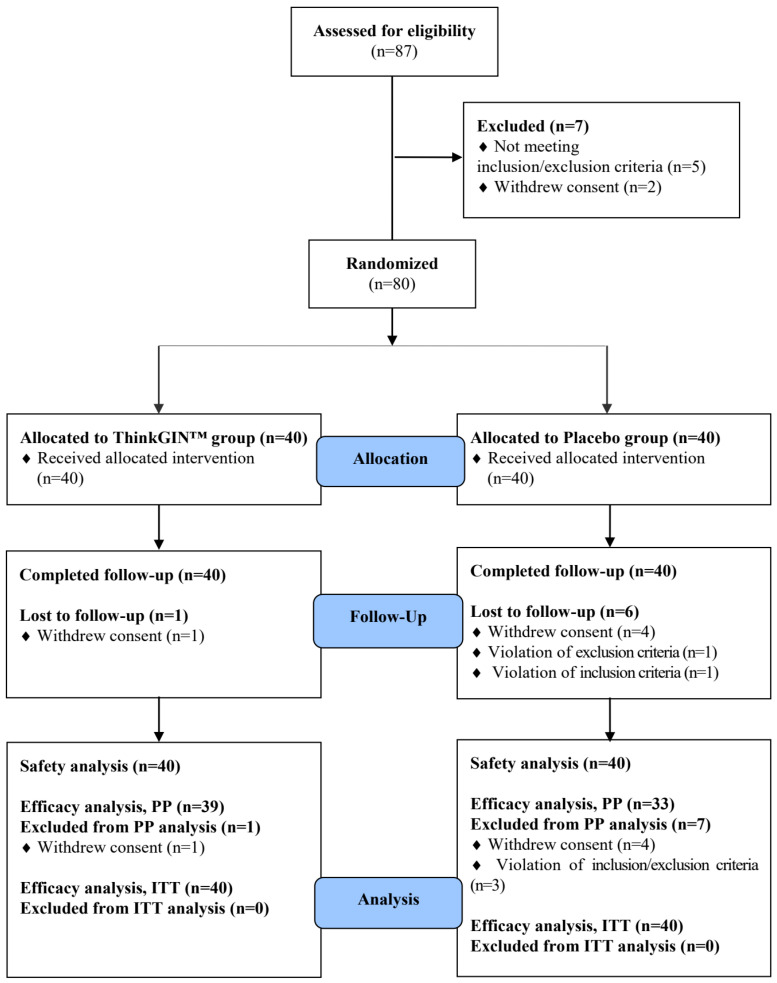
Flow-chart of subjects. Numbers of study participants enrolled, allocated, followed, and analyzed are shown using the CONSORT 2010 Flow Diagram.

A total of 87 volunteers participated in the screening test, and 80 subjects who met the inclusion/exclusion criteria were selected and randomly assigned to ThinkGIN™ and placebo groups. A total of 72 subjects (39 in the ThinkGIN™ group and 33 in the placebo group) completed all procedures according to the criteria of the protocol (Figure 2).

Demographic characteristics of all subjects are summarized in Table 1. Among subjects who participated in this study, there were 17 males and 63 females. Their average age was 63.20 ± 5.36 years, showing no statistically significant difference between the two groups (*p* > 0.05). There were no significant differences in education, height, weight, BMI, DBP, pulse, drinkers, or smokers between the two groups. However, the average SBP was 118.93 ± 11.25 mmHg in the ThinkGIN™ group and 126.00 ± 17.37 mmHg in the placebo group, showing a significant (*p* = 0.034) difference between the two groups. Additionally, as a result of analyzing lifestyle habits (product intake compliance, drinking and smoking amount survey, and so on), total intakes (*p* = 0.015) and compliance (*p* = 0.014) showed significant differences between the two groups in total. Therefore, statistical results were corrected for SBP, total intakes, and compliance and presented.

### 3.2. Dietary Intake and Physical Activity

To evaluate changes in dietary intake and physical activity during the study period, changes were assessed before and after 12 weeks of intervention. There was no significant difference in dietary intake (energy, carbohydrate, lipid, protein, fiber) between groups. Likewise, there was no statistically significant difference in physical activity (MET value) between ThinkGIN™ and placebo groups. Therefore, dietary intake and physical activity were well maintained during the study period without affecting study results.

### 3.3. Efficacy Outcomes

In the PP set, as a result of comparing baseline efficacy outcomes, there was a significant difference between groups in the language item (*p* = 0.011) of MoCA-K. Thus, statistical results were corrected by covariates and presented. In the ITT set, the total score (*p* = 0.026) and language items (*p* = 0.007) of the MoCA-K at baseline showed significant differences between groups. Thus, corrected statistical results were presented.

**Figure 3:**
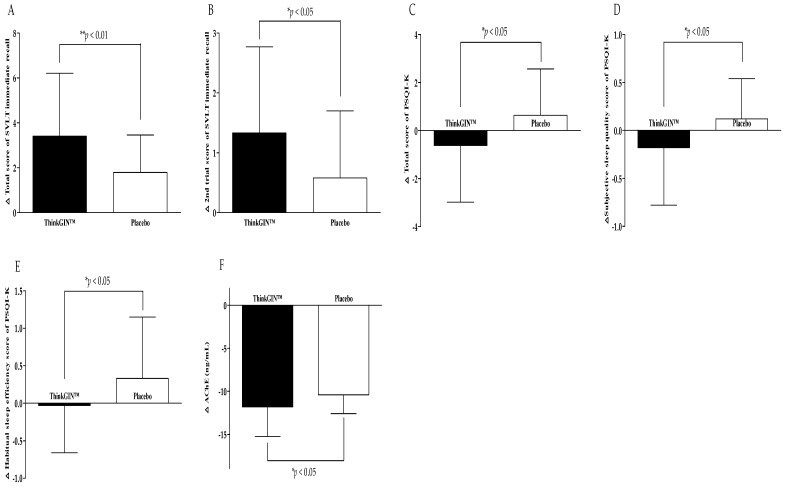
Changes in efficacy outcomes (PP analysis). (**A**) Total score of SVLT immediate recall, (**B**) 2nd trial score of SVLT immediate recall, (**C**) total score of PSQI-K, (**D**) subjective sleep quality score of PSQI-K, (**E**) habitual sleep efficiency score of PSQI-K, and (**F**) AChE were measured in ThinkGIN™ and placebo groups at baseline and 12 weeks. Values are presented as mean ± SD. Analyzed by independent *t*-test for change value between the groups. * *p* < 0.05, ** *p* < 0.01 vs. placebo group.

Efficacy biomarkers were measured before and at 12 weeks after the intervention. In the efficacy analysis on the PP set, changes in efficacy outcomes are summarized in Table 2 and Figure 3. After 12 weeks compared to baseline, the total score of SVLT immediate recall increased by 3.41 ± 2.80 in the ThinkGIN™ group and by 1.79 ± 1.67 in the placebo group, showing a statistically significant difference between the two groups (*p* = 0.004). The 2nd trial score of SVLT immediate recall increased by 1.33 ± 1.44 in the ThinkGIN™ group and 0.58 ± 1.12 in the placebo group, showing a statistically significant difference between the two groups (*p* = 0.016). Similar results were obtained when adjusted for covariates (*p* = 0.005 and *p* = 0.016, respectively). The total score of MoCA-K in the ThinkGIN™ group significantly increased by 1.33 ± 2.96 after 12 weeks of intake compared to baseline (*p* = 0.008), and showed a statistically significant trend compared to the placebo group (*p* = 0.068). Similar results were obtained upon correction (*p* = 0.078). After 12 weeks of intake compared to baseline, visuospatial-executive and language scores of MoCA-K increased in the ThinkGIN™ group but decreased in the placebo group, showing a significant trend between the two groups (*p* = 0.056, *p* = 0.096). The results were similar even after correction (*p* = 0.056, *p* = 0.068). As a result of analyzing PSQI-K before and 12 weeks after intake, the total score, subjective sleep quality, and habitual sleep efficiency scores decreased by 0.62 ± 2.36, 0.18 ± 0.60, and 0.03 ± 0.63, respectively, in the ThinkGIN™ group. In contrast, they increased by 0.64 ± 1.93, 0.12 ± 0.42, and 0.33 ± 0.82, respectively, in the placebo group, showing statistically significant differences between the two groups (*p* = 0.018, *p* = 0.015, and *p* = 0.039). Adjusted results were similar (*p* = 0.018, *p* = 0.018, and *p =* 0.039). The sleep latency score decreased in the ThinkGIN™ group but increased in the placebo group, showing a statistically significant trend between the two groups (*p* = 0.066). The results were the same even after correction. Blood AChE decreased by 11.83 ± 3.41 ng/mL in the ThinkGIN™ group and by 10.41 ± 2.19 ng/mL in the placebo group after 12 weeks compared to before intake. There was a statistically significant differences in change of blood AChE between the two groups (*p* = 0.037). Similar results were shown when adjusted (*p* = 0.044).

**Figure 4:**
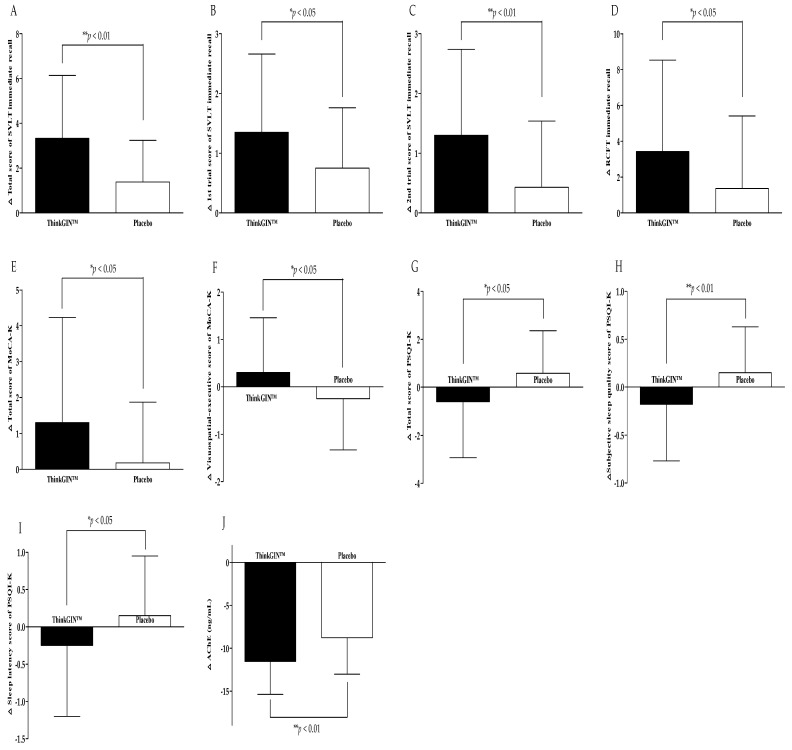
Changes in efficacy outcomes (ITT analysis). (**A**) Total score of SVLT immediate recall, (**B**) 1st trial score of SVLT immediate recall, (**C**) 2nd trial score of SVLT immediate recall, (**D**) RCFT immediate recall, (**E**) total score of MoCA-K, (**F**) visuospatial-executive score of MoCA-K, (**G**) total score of PSQI-K, (**H**) subjective sleep quality score of PSQI-K, (**I**) sleep latency score of PSQI-K, and (**J**) AChE were measured in ThinkGIN™ and placebo groups at baseline and 12 weeks. Values are presented as mean ± SD. Analyzed by independent *t*-test for change value between the groups. * *p* < 0.05, ** *p* < 0.01 vs. placebo group.

The ITT set was additionally analyzed. The results of efficacy evaluation are summarized in Table 3 and Figure 4. When comparing before and 12 weeks after intake, the total score, 1st trial, and 2nd trial scores of SVLT immediate recall increased by 3.33 ± 2.81, 1.35 ± 1.31, and 1.30 ± 1.44 in the ThinkGIN™ group, respectively. In the placebo group, they increased by 1.38 ± 1.86, 0.75 ± 1.01, and 0.43 ± 1.11, respectively. There were statistically significant differences between the two groups (*p* = 0.001, *p* = 0.024, and *p* = 0.003). The same results were obtained upon correction. The 3rd trial score of SVLT immediate recall in the ThinkGIN™ group significantly increased by 0.68 ± 1.38 after 12 weeks compared to baseline (*p* = 0.004). It showed a statistically significant trend compared to the placebo group (*p* = 0.092). The same results were obtained upon correction. The RCFT immediate recall score increased by 3.43 ± 5.10 in the ThinkGIN™ group and by 1.36 ± 4.05 in the placebo group. There was a statistically significant difference in change of RCFT immediate recall score between the two groups (*p* = 0.049). Adjusted results were the same. The RCFT recognition score in the ThinkGIN™ group significantly increased by 1.30 ± 2.29 after 12 weeks of intake compared to baseline (*p* = 0.001). It showed a statistically significant trend compared to the placebo group (*p* = 0.062). The results were the same even after correction. Changes from baseline to 12 weeks in the total score of MoCA-K increased statistically significantly to 1.30 ± 2.93 in the ThinkGIN™ group (*p* = 0.008). It was statistically significant when compared to the placebo group (*p* = 0.039). Similar results were obtained even when adjusted (*p* = 0.021). The visuospatial-executive score of MoCA-K increased by 0.30 ± 1.16 in the ThinkGIN™ group but decreased by 0.25 ± 1.08 in the placebo group after 12 weeks compared to baseline. There was a statistically significant difference between the two groups (*p* = 0.031). The results were the same even after correction. The MoCA-K language score increased in the ThinkGIN™ group but decreased in the placebo group, showing a statistically significant trend when it was compared between the two groups (*p* = 0.090). The results were similar even after correction (*p* = 0.069). Regarding changes from baseline to 12 weeks, total score, subjective sleep quality, and sleep latency score of PSQI-K decreased by 0.60 ± 2.33, 0.18 ± 0.59, and 0.25 ± 0.95, respectively, in the ThinkGIN™ group. On the other hand, they increased by 0.58 ± 1.78, 0.15 ± 0.48, and 0.15 ± 0.80, respectively, in the placebo group, showing significant differences between the two groups (*p* = 0.013, *p* = 0.009, and *p* = 0.046). The same results were obtained upon correction. The habitual sleep efficiency score decreased in the ThinkGIN™ group but increased in the placebo group, showing a statistically significant trend when compared between the two groups (*p* = 0.055). Adjusted results were the same. Blood AChE decreased by 11.53 ± 3.85 ng/mL in the ThinkGIN™ group and 8.78 ± 4.25 ng/mL in the placebo group after 12 weeks compared to that at baseline. There was a significant difference between the two groups (*p* = 0.003). The results were the same even after correction.

### 3.4. Safety Outcomes

The safety of ThinkGIN™ was evaluated based on the safety set. During intervention, AEs occurred in 8 subjects (4 in the ThinkGIN™ group and 4 in the placebo group). There was no statistically significant difference in the incidence of AEs between the two groups (10% vs. 10%, *p* > 0.05). All AEs were mild. They were not related to the intake of study product. There were no serious adverse events.

Based on our results, during the 12-week study period, no statistically significant differences were observed in any biomarkers of laboratory tests (CBC, biochemistry, urinalysis) between the two intake groups (Table 4). In addition, safety evaluation biomarkers, including vital signs and electrocardiography, showed no significant differences between the two groups, revealing that the study product was safe.

## 4. Discussion

This is the first double-blind, randomized, controlled trial (RCT) in humans to show a memory improvement effect of ThinkGIN™ manufactured with a smart farm system in subjects with SMI. Administration of ThinkGIN™ 450 mg for 12 weeks showed significant changes in SVLT, RCFT, MoCA-K, PSQI-K, and AChE without any side effects, confirming its effect of improving memory.

Pathological, functional, and structural changes in the brain begin to occur 20 years before clinical signs of dementia appear [38]. Thus, early recognition and management of the disease are very important to improve the clinical course of dementia [39,40]. SMI is common in older adults. It is receiving increasing attention as a pre-MCI stage in clinical manifestations of AD [41]. In longitudinal cohort studies [42,43], SMI has been identified as a predictor of cognitive decline and dementia in elderly subjects without cognitive impairment [41]. Therefore, SMI may represent the first changes in memory and cognition, corresponding to very subtle changes in the pre-MCI stage of AD. Thus, it might be an important clinical step for improving pre-dementia and pre-MCI in AD [38]. Therefore, in this study, subjects were recruited by targeting SMI to evaluate the effect of ThinkGIN™ in the pre-MCI stage. Despite the increasing aging population, currently there are no proven effective agents that can be used to reverse or delay the progression of SMI [44]. Therefore, this study is meaningful by verifying the memory improvement effect of ThinkGIN™ in SMI, suggesting that ThinkGIN™ can be used as an alternative prevention strategy in SMI.

In this study, both verbal memory (SVLT immediate recall) and visual memory (RCFT immediate recall) were improved after a 12-week intake of ThinkGIN™. This is a particularly noteworthy and important result considering that verbal memory decline is the best neuropsychological predictor of AD progression and cognitive decline [45]. In a similar clinical study of RCT design in MCI subjects, [46], taking *P. ginseng* powder (3 g/day) for 6 months significantly changed RCFT immediate recall and delayed recall between groups, improved visual memory and visual learning. On the other hand, SVLT immediate recall showed no change. In our study, both verbal and visual memory were improved despite a relatively low dose of ThinkGIN™ (450 mg/day) and a short study period (12 weeks). A clinical study conducted with *P. ginseng* powder [46] contained a total 53 mg/g of ginsenosides, but ThinkGIN™ contained a total 103.32 mg/g of ginsenosides [17], which was higher. In a review paper on ginsenosides and cognitive function [47], the effect of improving memory and cognitive function was shown by ginsenosides regulating signaling pathways related to oxidative stress, apoptosis, inflammation, synaptic plasticity, and neurogenesis. Therefore, ThinkGIN™ has a higher ginsenoside content than regular ginseng, so its memory improvement effect is more comprehensive.

MoCA is the most widely used tool to evaluate the degree of cognitive impairment [48]. A similar previous study [49] has shown that taking Huannao Yicong formula (5 g/day) containing ginseng for 6 months can significantly increase MoCA of mild to moderate AD patients compared to the control. In this study, the MoCA-K total score was significantly increased when ThinkGIN™ (450 mg/day) was consumed for 12 weeks in semi-healthy individuals (SMI), not patients. This proves that overall cognitive function, including memory, can be improved with a single, low dose, short-term intake of ThinkGIN™.

Sleep disorders are common in the elderly. Aging tends to decrease deep sleep. Thus, the elderly tend to wake up frequently and wake up early in the morning [50]. For this reason, sleep has an important physiological impact on memory. Sleep disturbance can cause subjective or objective memory impairment regardless of brain pathology [51]. Therefore, in this study, the PSQI-K questionnaire was used to examine the effect of ThinkGIN™ on sleep quality. If the PSQI total score exceeds 5 points, that person is defined as a poor sleeper. In this study, the baseline PSQI total score exceeded 5 points. Thus, subjects with SMI and poor sleep quality participated in this study. Consumption of ThinkGIN™ for 12 weeks significantly improved the PSQI-K total score, subjective sleep quality, habitual sleep efficiency, and sleep latency. In previous clinical trials [52,53,54], administration of red ginseng powder and fermented red ginseng improved sleep time and sleep efficiency, similar to our results. ThinkGIN™ has an advantage for commercialization because it is easy to supply through standardized cultivation. In addition, it can reduce costs with a short cultivation period.

Because AChE decomposes acetylcholine (ACh), a neurotransmitter, it can cause decline in cognitive function and memory [55]. Accordingly, AChE inhibitors are used to treat dementia [56]. However, all AChE inhibitors show dose-dependent side effects, mainly in the digestive system [57,58,59]. Therefore, there is a need to develop functional foods to replace AChE inhibitors [60]. In this study, blood AChE levels were statistically significantly reduced after consumption of ThinkGIN™ for 12 weeks compared to those in the placebo group. In addition, ThinkGIN™ had no side effects. In a previous study conducted with hydroponic ginseng sprout extract [61], AChE activity was decreased by ginseng sprout extract, suggesting that it could improve memory by increasing ACh, a neurotransmitter important for regulating synaptic plasticity. A previous study [62] has also shown that ginseng extract can increase ACh synthesis and inhibit ACh degradation, which can significantly increase synaptic ACh levels and improve scopolamine-induced amnesia. These results indicate that ThinkGIN™ can significantly reduce AChE, thereby improving memory by enhancing the production of ACh.

Preclinical studies have suggested that ginseng sprouts can improve memory [17,31,61]. In a scopolamine-induced memory deficit mouse model [17], ThinkGIN™ regulated synaptic plasticity by activating the signaling pathway of AKT/ERK/CREB and reduced cortical inflammation by inhibiting COX-2, NLRP3, and Iba-1, thereby improving the memory deficit behavior. In addition, ThinkGIN™ can significantly suppress inflammatory responses by regulating the production of iNOS, COX-2, and NO in BV-2 microglial cells induced by LPS. HPLC confirmed that production of sprout ginseng through smart farms can increase the content of ginsenosides (Rb2, Rb1, Rc, Rd, and Re). It has been suggested that ginsenosides can improve memory by improving neuroinflammation [17]. In vitro studies [61] of hydroponic sprout ginseng extract have shown that it can inhibit neurotoxicity (caspase-3, PPAR, Bax, Bcl-2), decrease neuroinflammatory responses (MAPK, NF-κB p65, and COX-2), and regulate synaptic plasticity. It also showed an effect of improving memory through CREB activation (BDNF) and neurotransmitter regulation (AChE). In addition, ginseng sprouts (70% ethanol extract of ginseng sprouts manufactured by Smart Farm) and Kyung-Ok-Ko mixture showed memory effects in animal behavioral tests in a scopolamine-induced memory impairment mouse model [31]. Ginsenoside Re, which has the highest content in sprout ginseng extract, has also been reported to improve memory based on preclinical tests. It is attracting attention as a major active ingredient in improving memory. In aged Klotho deficient mice, ginsenoside Re can improve cognition and memory through regulation of Nrf2/GPx-1/ERK/CREB signaling, AT1 receptor expression, and NOX-, ROS-, and GPx- levels [63]. In addition, ginsenoside Re reduces Aβ production by inhibiting β-secretase 1 (BACE1) activity [64]. It can also alleviate cholinergic deficits by activating the cholinergic neurotransmitter system [65]. Therefore, this clinical trial was conducted using ThinkGIN™ with increased ginsenoside content through smart farming. Preclinical results suggest that it has a memory-improving effect through modulation of synaptic plasticity, neuroinflammatory effects, and neurotransmitter regulation.

The progression of SMI and MCI to dementia can take several years to even 10 years [39,66,67]. Thus, prevention and treatment products must be safe with no harmful effects when used long-term [68]. *P. ginseng* is one of the most frequently used herbs worldwide [69]. *P. ginseng* has been consumed for a long time. It is generally reported to have a good safety profile and low incidence of side effects [70]. Recently, ginseng sprouts have been established as a safe and new medicinal vegetable according to the revised Ginseng Industry Act 2015 [71]. The interest in ginseng sprouts is increasing [72]. Electroconvulsive therapy (ECT) has been reported to have cognitive effects in patients with major depressive disorder [73,74]. ECT uses an electric current to induce brief seizures in the brain, resulting in quick and strong effects [75]. However, ECT is associated with loss of autobiographical memory and short-term decline in new learning [76]; therefore, ECT treatment is controversial because its effectiveness comes with a risk of side effects. On the other hand, in this study, a 12-week intake of ThinkGIN™ was safe in SMI subjects as no clinically significant adverse reactions or changes in safety biomarkers (laboratory tests, vital signs, electrocardiogram, etc.) were observed. As a result, we conclude ginseng sprouts improve memory without side effects and are a safe and promising functional food alternative to ECT. Therefore, ThinkGIN™ may be a potential candidate for long-term use in SMI, a precursor to dementia and MCI.

*P. ginseng* is known to have various effects. However, it has the disadvantage in that it takes a long time to grow. In addition, it is exposed to pests and diseases during that time, making the use of pesticides inevitable [77]. To compensate for this disadvantage, ginseng sprouts grown in a smart farm system can be cultivated within a relatively short period of time. It has the advantage that all roots, stems, and leaves can be consumed. It has also been reported that the content of ginsenosides, the main active ingredients in ginseng, is higher in ginseng sprouts [17,18,21,78]. Two clinical studies [79,80] reported that ginseng had a good effect on cognitive improvement in AD patients. These results are similar to those of our study. However, the two clinical studies targeted patients and were designed as open-label trials, so there are limitations. Therefore, this clinical study targeted SMI and was conducted with the design of a randomized, double-blind, placebo-controlled clinical trial. Recently, preclinical research on memory and cognition of ginseng sprouts has been conducted. However, there is no research on humans. Therefore, this study is meaningful in that it is the first clinical study of ginseng sprout targeting humans with an RCT design.

Nevertheless, this study has several potential limitations. First, the sample size was relatively small. Although the sample size was scientifically calculated based on previous research, additional research on a larger scale is needed to resolve the issue of generalizability of research results. Second, the intake period was limited to 12 weeks. Due to the relatively short study period, there was a possibility of improvement in the placebo group due to the learning effect affecting neuropsychological test results. However, fortunately, the memory improvement effect in the ThinkGIN™ group was sufficiently strong compared to that in the placebo group, with statistical significance between groups confirmed. Additionally, since long-term intake and residual effects of ThinkGIN™ on memory improvement could not be confirmed, future studies should increase the study period and follow-up visits. Third, the biomarkers used in this study were limited to memory assessment tools, subjective questionnaires, and blood tests. Previous studies [81] have shown that combining magnetic resonance imaging (MRI) with other biomarkers is expected to have the greatest prognostic value in the early stages of AD. Therefore, future studies need to measure various markers, such as MRI, to more accurately evaluate the memory improvement effect. Fourth, the intake time was limited to morning only. According to the pharmacokinetics study of ginsenoside Re [82], the T_max_ of ginsenoside Re was 1.19 ± 0.44 h after oral intake, so it was taken only in the morning to maximize bioavailability throughout the day and maintain a consistent routine for the subjects. However, in clinical studies on insomnia [83,84], it was reported that taking it before bedtime was effective in improving sleep quality and satisfaction. Therefore, future research is needed based on various intake times. Finally, although sex is considered an important parameter in estimating the risk of AD disease [85], the number of males participating in the study was relatively small compared to that of females (male: 21.25%, female: 78.75%). Fortunately, there was no significant difference in sex ratio between groups. In addition, ThinkGIN™ provided important insight into the effect of improving memory.

Our study is the first well-designed clinical trial to evaluate the efficacy and safety of standardized ThinkGIN™ in improving memory. ThinkGIN™ is safe without any side effects. It provides sufficient evidence for memory improvement.

## 5. Conclusions

A 12-week, randomized, double-blind, placebo-controlled clinical trial was conducted to evaluate the efficacy and safety of ThinkGIN™ for memory improvement in SMI subjects. We found that oral intake of ThinkGIN™ for 12 weeks significantly improved verbal memory, visual memory, overall cognitive function, sleep quality, and blood AChE compared to the placebo. We also confirmed its safety. Therefore, this study provides sufficient clinical evidence that ThinkGIN™ manufactured in a smart farm system can be used as a functional food to improve memory.

## Figures and Tables

**Table 1:** Baseline demographic characteristics of subjects.

	ThinkGIN™ Group (*n* = 40)	Placebo Group (*n* = 40)	Total (*n* = 80)	*p*-Value ^1^
Sex (M/F)	7/33	10/30	17/63	0.412 ^2^
Age (years)	63.45 ± 5.05	62.95 ± 5.71	63.20 ± 5.36	0.679
Education (years)	12.63 ± 2.91	13.40 ± 3.18	13.01 ± 3.05	0.259
Height (cm)	157.93 ± 6.68	160.48 ± 8.52	159.20 ± 7.72	0.140
Weight (kg)	59.76 ± 9.70	63.54 ± 10.81	61.65 ± 10.38	0.104
BMI (kg/m^2^)	23.86 ± 2.72	24.55 ± 2.82	24.21 ± 2.78	0.267
SBP (mmHg)	118.93 ± 11.25	126.00 ± 17.37	122.46 ± 14.97	0.034 *
DBP (mmHg)	74.53 ± 8.02	76.18 ± 8.99	75.35 ± 8.51	0.389
Pulse (beats/minute)	74.28 ± 7.63	74.23 ± 10.06	74.25 ± 8.87	0.980
Alcohol (*n*, %)	11 (27.50)	13 (32.50)	24 (30.00)	0.626 ^2^
Alcohol (units/week)	4.44 ± 6.45	4.31 ± 3.60	4.37 ± 4.98	0.951
Smoking (*n*, %)	2 (5.00)	3 (7.50)	5 (6.25)	1.000 ^3^

Values are presented as mean ± SD or number (%). ^1^ Analyzed by independent *t*-test between the groups. ^2^ Analyzed by chi-square test between the groups. ^3^ Analyzed by Fisher’s exact test between the groups. * *p* < 0.05.

**Table 2:** Changes in efficacy outcomes before and after 12 weeks of intake (PP analysis).

			ThinkGIN™ (*n* = 39)	Placebo (*n* = 33)	*p*-Value ^1^	*Adj.p*-Value ^2^
SVLT	immediate recall	total score	3.41 ± 2.80	1.79 ± 1.67	0.004 **	0.005 **
		1st trial	1.38 ± 1.31	0.94 ± 1.00	0.115	0.115
		2nd trial	1.33 ± 1.44	0.58 ± 1.12	0.016 *	0.016 *
		3rd trial	0.69 ± 1.40	0.27 ± 1.18	0.178	0.178
	delayed recall		0.92 ± 1.75	0.39 ± 1.32	0.159	0.159
	recognition		0.90 ± 2.36	0.27 ± 1.94	0.229	0.229
RCFT	Copy	copy score	−1.26 ± 2.60	−1.45 ± 3.39	0.780	0.780
		copy time (s)	−15.03 ± 67.07	−37.64 ± 83.91	0.208	0.208
	immediate recall		3.51 ± 5.14	1.70 ± 4.39	0.115	0.115
	delayed recall		2.46 ± 4.76	1.45 ± 4.38	0.357	0.357
	recognition		1.33 ± 2.31	0.52 ± 2.00	0.116	0.116
MoCA-K	total score		1.33 ± 2.96	0.27 ± 1.82	0.068	0.078
	visuospatial-executive		0.31 ± 1.17	−0.21 ± 1.08	0.056	0.056
	naming		0.00 ± 0.65	0.06 ± 0.70	0.705	0.705
	attention		0.05 ± 0.97	0.06 ± 0.70	0.964	0.964
	language		0.13 ± 0.57	−0.09 ± 0.52	0.096	0.068 ^3^
	abstraction		−0.03 ± 0.71	0.09 ± 0.38	0.379	0.400
	delayed recall		0.72 ± 1.62	0.30 ± 1.47	0.263	0.263
	orientation		0.15 ± 0.84	0.06 ± 0.50	0.563	0.572
SMCQ			−1.08 ± 2.07	−0.61 ± 1.41	0.258	0.272
PSQI-K	total score		−0.62 ± 2.36	0.64 ± 1.93	0.018 *	0.018 *
	subjective sleep quality		−0.18 ± 0.60	0.12 ± 0.42	0.015 *	0.018 *
	sleep latency		−0.26 ± 0.97	0.15 ± 0.87	0.066	0.066
	sleep duration		−0.03 ± 0.74	0.24 ± 0.90	0.171	0.171
	habitual sleep efficiency		−0.03 ± 0.63	0.33 ± 0.82	0.039 *	0.039 *
	sleep disturbances		−0.05 ± 0.46	−0.03 ± 0.39	0.837	0.837
	use of sleeping medication		0.00 ± 0.00	0.00 ± 0.00	-	-
	daytime dysfunction		−0.08 ± 0.81	−0.18 ± 0.88	0.600	0.600
Blood biomarkers related to memory	AChE (ng/mL)		−11.83 ± 3.41	−10.41 ± 2.19	0.037 *	0.044 *
	BDNF (pg/mL)		19,178.44 ± 10,716.94	21,133.00 ± 9689.28	0.423	0.423
	amyloid β 1–40 (pg/mL)		4.82 ± 181.31	23.43 ± 37.56	0.535	0.565
	amyloid β 1–42 (pg/mL)		3.71 ± 13.09	2.17 ± 2.25	0.476	0.509
	amyloid β 40/42		−63.34 ± 126.53	−64.77 ± 110.46	0.960	0.960
	ApoE4 (pg/mL)		7858.05 ± 199,152.49	−45,397.27 ± 173,563.04	0.235	0.235
TAS (mmol/L)			−0.76 ± 0.27	−0.85 ± 0.29	0.169	0.169
hs-CRP (mg/L)			0.50 ± 2.85	0.65 ± 1.39	0.767	0.778

Values are presented as mean ± SD. ^1^ Analyzed by independent *t*-test for change value between the groups. ^2^ Analyzed by ANCOVA (adjusted on SBP, total intakes, compliance). ^3^ Analyzed by ANCOVA (adjusted on SBP, total intakes, compliance, baseline of MoCA-K (language)). * *p* < 0.05, ** *p* < 0.01 vs. placebo group.

**Table 3:** Changes in efficacy outcomes before and after 12 weeks of intake (ITT analysis).

			ThinkGIN™ (*n* = 40)	Placebo (*n* = 40)	*p*-Value ^1^	*Adj. p*-Value ^2^
SVLT	immediate recall	total score	3.33 ± 2.81	1.38 ± 1.86	0.001 **	0.001 **
		1st trial	1.35 ± 1.31	0.75 ± 1.01	0.024 *	0.024 *
		2nd trial	1.30 ± 1.44	0.43 ± 1.11	0.003 **	0.003 **
		3rd trial	0.68 ± 1.38	0.20 ± 1.09	0.092	0.092
	delayed recall		0.90 ± 1.74	0.35 ± 1.21	0.105	0.104
	recognition		0.88 ± 2.33	0.20 ± 1.77	0.149	0.149
RCFT	Copy	copy score	−1.23 ± 2.57	−1.13 ± 3.19	0.878	0.878
		copy time (s)	−14.65 ± 66.25	−31.65 ± 77.23	0.294	0.294
	immediate recall		3.43 ± 5.10	1.36 ± 4.05	0.049 *	0.049 *
	delayed recall		2.40 ± 4.72	1.23 ± 4.00	0.233	0.233
	recognition		1.30 ± 2.29	0.43 ± 1.82	0.062	0.062
MoCA-K	total score		1.30 ± 2.93	0.18 ± 1.69	0.039 *	0.021 *^3^
	visuospatial-executive		0.30 ± 1.16	−0.25 ± 1.08	0.031 *	0.031 *
	naming		0.00 ± 0.64	0.05 ± 0.64	0.728	0.728
	attention		0.05 ± 0.96	0.05 ± 0.64	1.000	1.000
	language		0.13 ± 0.56	−0.08 ± 0.47	0.090	0.069 ^4^
	abstraction		−0.03 ± 0.70	0.05 ± 0.39	0.555	0.554
	delayed recall		0.70 ± 1.60	0.30 ± 1.36	0.233	0.233
	orientation		0.15 ± 0.83	0.05 ± 0.45	0.507	0.506
SMCQ			−1.05 ± 2.05	−0.53 ± 1.30	0.176	0.175
PSQI-K	total score		−0.60 ± 2.33	0.58 ± 1.78	0.013 *	0.013 *
	subjective sleep quality		−0.18 ± 0.59	0.15 ± 0.48	0.009 **	0.009 **
	sleep latency		−0.25 ± 0.95	0.15 ± 0.80	0.046 *	0.046 *
	sleep duration		−0.03 ± 0.73	0.20 ± 0.82	0.201	0.201
	habitual sleep efficiency		−0.03 ± 0.62	0.28 ± 0.75	0.055	0.055
	sleep disturbances		−0.05 ± 0.45	−0.03 ± 0.36	0.784	0.784
	use of sleeping medication		0.00 ± 0.00	0.00 ± 0.00	-	-
	daytime dysfunction		−0.08 ± 0.80	−0.18 ± 0.81	0.580	0.580
Blood biomarkers related to memory	AChE (ng/mL)		−11.53 ± 3.85	−8.78 ± 4.25	0.003 **	0.003 **
	BDNF (pg/mL)		18,698.98 ± 11,004.69	18,317.18 ± 11,947.91	0.882	0.882
	amyloid β 1–40 (pg/mL)		4.69 ± 178.97	16.60 ± 40.56	0.684	0.683
	amyloid β 1–42 (pg/mL)		3.62 ± 12.94	1.79 ± 2.20	0.385	0.383
	amyloid β 40/42		−61.76 ± 125.29	−54.54 ± 102.76	0.779	0.779
	ApoE4 (pg/mL)		7661.60 ± 196,586.60	−37,401.80 ± 158,197.42	0.262	0.262
TAS (mmol/L)			−0.75 ± 0.29	−0.73 ± 0.40	0.824	0.824
hs-CRP (mg/L)			0.48 ± 2.81	0.53 ± 1.29	0.928	0.927

Values are presented as mean ± SD. ^1^ Analyzed by independent *t*-test for change value between the groups. ^2^ Analyzed by ANCOVA (adjusted on SBP, total intakes, compliance). ^3^ Analyzed by ANCOVA (adjusted on SBP, total intakes, compliance, baseline of MoCA-K (total score)). ^4^ Analyzed by ANCOVA (adjusted on SBP, total intakes, compliance, baseline of MoCA-K (language)). * *p* < 0.05, ** *p* < 0.01 vs. placebo group.

**Table 4:** Changes in laboratory tests before and after 12 weeks of intake (safety analysis).

		ThinkGIN™ (*n* = 40)				Placebo (*n* = 40)				*p*-Value ^2^
		Baseline	12 Week	Change Value	*p*-Value ^1^	Baseline	12 Week	Change Value	*p*-Value ^1^	
CBC	Hemoglobin (g/dL)	13.54 ± 1.17	13.36 ± 1.09	−0.18 ± 0.67	0.103	13.90 ± 1.31	13.67 ± 1.24	−0.23 ± 0.48	0.005 **	0.711
	Hematocrit (%)	40.15 ± 3.54	39.43 ± 3.22	−0.72 ± 2.18	0.043 *	41.30 ± 4.01	40.51 ± 3.88	−0.79 ± 1.72	0.006 **	0.883
	WBC (K/UL)	5.22 ± 1.16	5.15 ± 1.32	−0.07 ± 1.05	0.690	5.56 ± 1.23	5.42 ± 1.19	−0.13 ± 0.76	0.274	0.747
	RBC (M/UL)	4.31 ± 0.36	4.32 ± 0.36	0.01 ± 0.22	0.717	4.39 ± 0.43	4.40 ± 0.42	0.01 ± 0.19	0.729	0.970
	Platelet (K/UL)	231.48 ± 61.45	215.60 ± 56.88	−15.88 ± 26.72	0.001 **	228.43 ± 38.36	220.13 ± 41.38	−8.30 ± 34.46	0.136	0.275
Biochemistry	AST (U/L)	25.03 ± 6.20	24.63 ± 6.50	−0.40 ± 5.48	0.647	25.08 ± 6.64	24.53 ± 5.63	−0.55 ± 4.42	0.436	0.893
	ALT (U/L)	21.55 ± 10.19	21.65 ± 12.34	0.10 ± 8.96	0.944	24.20 ± 9.40	22.95 ± 8.61	−1.25 ± 5.62	0.168	0.422
	Gamma-GT (U/L)	19.60 ± 13.36	19.80 ± 19.45	0.20 ± 10.85	0.908	28.55 ± 34.35	26.80 ± 27.55	−1.75 ± 11.86	0.357	0.445
	Total cholesterol (mg/dL)	206.20 ± 48.85	199.65 ± 48.20	−6.55 ± 30.31	0.180	203.15 ± 37.92	202.15 ± 35.22	−1.00 ± 33.11	0.850	0.437
	LDL-C (mg/dL)	124.58 ± 36.16	123.68 ± 35.24	−0.90 ± 24.13	0.815	123.53 ± 35.09	122.45 ± 31.00	−1.08 ± 27.98	0.809	0.976
	HDL-C (mg/dL)	61.35 ± 19.18	58.08 ± 15.30	−3.28 ± 10.55	0.057	59.58 ± 13.47	57.25 ± 13.64	−2.33 ± 7.92	0.071	0.650
	Albumin (g/dL)	4.28 ± 0.27	4.26 ± 0.27	−0.02 ± 0.27	0.641	4.22 ± 0.27	4.32 ± 0.19	0.10 ± 0.31	0.057	0.078
	Total protein (g/dL)	7.25 ± 0.36	7.19 ± 0.29	−0.05 ± 0.33	0.316	7.16 ± 0.35	7.25 ± 0.31	0.09 ± 0.35	0.132	0.073
	Total bilirubin (mg/dL)	0.98 ± 0.26	0.94 ± 0.26	−0.04 ± 0.20	0.248	0.97 ± 0.22	0.87 ± 0.24	−0.10 ± 0.17	0.001 **	0.151
	ALP (U/L)	61.78 ± 14.99	63.80 ± 17.24	2.03 ± 12.19	0.300	61.98 ± 16.04	67.05 ± 14.64	5.08 ± 8.88	0.001 **	0.205
	LD (U/L)	171.00 ± 35.93	160.30 ± 28.61	−10.70 ± 30.00	0.030 *	169.05 ± 34.09	163.78 ± 27.33	−5.28 ± 18.74	0.083	0.336
	Glucose (mg/dL)	101.85 ± 7.87	96.95 ± 11.08	−4.90 ± 9.65	0.003 **	104.80 ± 11.11	99.68 ± 9.96	−5.13 ± 8.49	0.001 **	0.912
	BUN (mg/dL)	16.45 ± 3.29	15.53 ± 3.02	−0.92 ± 2.78	0.043 *	14.97 ± 2.76	14.55 ± 3.12	−0.42 ± 2.58	0.307	0.410
	Triglyceride (mg/dL)	117.45 ± 71.58	115.63 ± 70.09	−1.83 ± 40.41	0.777	127.20 ± 56.69	130.50 ± 51.37	3.30 ± 46.37	0.655	0.600
	Creatinine (mg/dL)	0.85 ± 0.17	0.83 ± 0.15	−0.02 ± 0.10	0.233	0.83 ± 0.15	0.84 ± 0.14	0.01 ± 0.09	0.458	0.168
	CK (U/L)	136.93 ± 69.62	137.55 ± 82.88	0.63 ± 72.48	0.957	120.58 ± 110.47	108.08 ± 65.52	−12.50 ± 92.37	0.397	0.482
Urin-alysis	pH	6.45 ± 1.07	6.44 ± 1.03	−0.01 ± 0.92	0.932	6.54 ± 1.00	6.53 ± 0.94	−0.01 ± 1.15	0.945	1.000
	Specific gravity	1.02 ± 0.01	1.02 ± 0.01	0.00 ± 0.01	0.324	1.02 ± 0.01	1.02 ± 0.01	0.00 ± 0.01	0.529	0.262

Values are presented as mean ± SD. ^1^ Analyzed by paired *t*-test between baseline and 12 weeks within each group. ^2^ Analyzed by independent *t*-test for change value between the groups. * *p* < 0.05, ** *p* < 0.01 vs. baseline.

## Data Availability

The data presented in this study are available within the article.
